# Atualização da Diretriz de Avaliação Cardiovascular Perioperatória da Sociedade Brasileira de Cardiologia: Foco em Manejo dos Pacientes com Intervenção Coronária Percutânea – 2022

**DOI:** 10.36660/abc.20220039

**Published:** 2022-02-14

**Authors:** Daniela Calderaro, Luciana Dornfeld Bichuette, Pamela Camara Maciel, Francisco Akira Malta Cardozo, Henrique Barbosa Ribeiro, Danielle Menosi Gualandro, Luciano Moreira Baracioli, Alexandre de Matos Soeiro, Carlos Vicente Serrano, Ricardo Alves da Costa, Bruno Caramelli

**Affiliations:** 1 Incor Hospital das Clínicas HCFMUSP São Paulo SP Brasil Instituto do Coração (Incor) do Hospital das Clínicas da Faculdade de Medicina da Universidade de São Paulo (HCFMUSP), São Paulo, SP – Brasil; 2 CRIB University Hospital Basel University of Basel Basel Suíça Department of Cardiology and Cardiovascular Research Institute Basel (CRIB), University Hospital Basel, University of Basel, Basel – Suíça; 3 Hospital Sírio Libanês São Paulo SP Brasil Hospital Sírio Libanês, São Paulo, SP – Brasil; 4 Hospital BP Mirante São Paulo SP Brasil Hospital BP Mirante, São Paulo, SP – Brasil; 5 Instituto Dante Pazzanese de Cardiologia São Paulo SP Brasil Instituto Dante Pazzanese de Cardiologia, São Paulo, SP – Brasil

**Table:** 

Atualização da Diretriz de Avaliação Cardiovascular Perioperatória da Sociedade Brasileira de Cardiologia: Foco em Manejo dos Pacientes com Intervenção Coronária Percutânea – 2022	
O relatório abaixo lista as declarações de interesse conforme relatadas à SBC pelos especialistas durante o período de desenvolvimento deste posicionamento, 2021.	
Especialista	Tipo de relacionamento com a indústria
Alexandre de Matos Soeiro	Financiamento de atividades de educação médica continuada, incluindo viagens, hospedagens e inscrições para congressos e cursos, provenientes da indústria farmacêutica, de órteses, próteses, equipamentos e implantes, brasileiras ou estrangeiras: - Bayer, Pfizer, Daiichi Sankyo, Biolab.
Bruno Caramelli	Nada a ser declarado
Carlos Vicente Serrano Jr.	Nada a ser declarado
Daniela Calderaro	Declaração financeira A - Pagamento de qualquer espécie e desde que economicamente apreciáveis, feitos a (i) você, (ii) ao seu cônjuge/ companheiro ou a qualquer outro membro que resida com você, (iii) a qualquer pessoa jurídica em que qualquer destes seja controlador, sócio, acionista ou participante, de forma direta ou indireta, recebimento por palestras, aulas, atuação como proctor de treinamentos, remunerações, honorários pagos por participações em conselhos consultivos, de investigadores, ou outros comitês, etc. Provenientes da indústria farmacêutica, de órteses, próteses, equipamentos e implantes, brasileiras ou estrangeiras: - Bayer: Xarelto e Finerinone; Janssen: hipertensão pulmonar Outros relacionamentos Financiamento de atividades de educação médica continuada, incluindo viagens, hospedagens e inscrições para congressos e cursos, provenientes da indústria farmacêutica, de órteses, próteses, equipamentos e implantes, brasileiras ou estrangeiras: - Bayer: Xarelto; Daiichi-Sankyo: Lixiana; Janssen: hipertensão pulmonar
Danielle Menosi Gualandro	Nada a ser declarado
Francisco Akira Malta Cardozo	Declaração financeira A - Pagamento de qualquer espécie e desde que economicamente apreciáveis, feitos a (i) você, (ii) ao seu cônjuge/ companheiro ou a qualquer outro membro que resida com você, (iii) a qualquer pessoa jurídica em que qualquer destes seja controlador, sócio, acionista ou participante, de forma direta ou indireta, recebimento por palestras, aulas, atuação como proctor de treinamentos, remunerações, honorários pagos por participações em conselhos consultivos, de investigadores, ou outros comitês, etc. Provenientes da indústria farmacêutica, de órteses, próteses, equipamentos e implantes, brasileiras ou estrangeiras: - Bayer: Xarelto. C - Financiamento de pesquisa (pessoal), cujas receitas tenham sido provenientes da indústria farmacêutica, de órteses, próteses, equipamentos e implantes, brasileiras ou estrangeiras. - Bayer: Xarelto. Outros relacionamentos Financiamento de atividades de educação médica continuada, incluindo viagens, hospedagens e inscrições para congressos e cursos, provenientes da indústria farmacêutica, de órteses, próteses, equipamentos e implantes, brasileiras ou estrangeiras: - Bayer: Xarelto.
Henrique Barbosa Ribeiro	Nada a ser declarado
Luciana Dornfeld Bichuette	Nada a ser declarado
Luciano Moreira Baracioli	Outros relacionamentos Financiamento de atividades de educação médica continuada, incluindo viagens, hospedagens e inscrições para congressos e cursos, provenientes da indústria farmacêutica, de órteses, próteses, equipamentos e implantes, brasileiras ou estrangeiras: - Bayer: Xarelto.
Pamela Camara Maciel	Nada a ser declarado
Ricardo Alves da Costa	Nada a ser declarado

## 1. Introdução

O cuidado de pacientes submetidos à intervenção coronária percutânea (ICP) e à eventual necessidade de cirurgia não cardíaca precoce é um dos tópicos que gera mais debate em medicina perioperatória, pois envolve questões importantes sobre o manejo da terapia antitrombótica, além das questões habituais de manejo do risco cardíaco em coronariopatia. Se, por um lado, é necessário lidar com o risco hemorrágico, inerente à cirurgia e potencializado pelos antiagregantes plaquetários, por outro, é fundamental considerar o aumento do risco de trombose de *stent* , especialmente se o tempo programado de dupla antiagregação plaquetária (DAPT) for abreviado. Elementos como premência e porte do procedimento cirúrgico proposto, estado clínico do paciente e dados referentes à angioplastia coronária, tais como intervalo decorrido, contexto eletivo ou de urgência, resultado primário obtido e tipo de *stent* utilizado, são fundamentais para a individualização das recomendações, ponderando-se o risco hemorrágico ( Tabela 1 ) e o risco trombótico ( Tabela 2 ).

**Tabela 1:** – Fatores associados a risco elevado de sangramento1-6

1) Fatores associados a risco elevado de sangramento
**Fatores inerentes ao procedimento cirúrgico**
**Baixo risco:**
- Procedimentos gastrointestinais (p. ex., endoscopia, colonoscopia e cápsula endoscópica)
- Procedimentos cardiovasculares (p. ex., implante de marca-passo/CDI ou troca de gerador, ablação cardíaca, cateterismo coronariano por via radial)
- Procedimentos dermatológicos (p. ex., biópsia de pele)
- Procedimentos oftalmológicos (p. ex., cirurgia de catarata)
- Procedimento odontológicos (p. ex., extração de até dois dentes e procedimento endodôntico)
**Alto risco:**
- Cirurgias com necessidade de anestesia neuroaxial
- Neurocirurgias
- Cirurgias vasculares maiores (p. ex., correção de aneurisma de aorta e endarterectomia carotídea)
- Cirurgias abdominais de grande porte (p. ex., cirurgias de ressecção neoplásica, ressecção de doença diverticular ou inflamatórias intestinais)
- Cirurgias ortopédicas maiores (p. ex., artroplastia de quadril e joelho)
**2) Fatores clínicos**
História de sangramento prévio
Uso de anticoagulantes orais
Sexo feminino
Idade avançada
Doença renal crônica
Diabetes melito
Anemia
Plaquetopenia
Uso crônico de corticosteroides ou anti-inflamatórios não esteroides

*CDI: cardiodesfibrilador implantável.*

**Tabela 2:** – Fatores de risco associados a trombose de stent e eventos isquêmicos pós-angioplastia7-12

Fatores de risco associados à trombose de stent
Suspensão precoce da DAPT
Síndrome coronariana aguda
Diabetes melito
Tabagismo
Neoplasia
Doença arterial periférica
Stent farmacológico de primeira geração
Fração de ejeção ventricular <40%
Lesão de descendente anterior proximal
Angioplastia prévia
Stent em bifurcação coronariana
Stent de pequeno diâmetro
Reestenose intrastent
Stent subdimensionado
Stent de longo comprimento
**Fatores de risco associados a eventos isquêmicos pós-angioplastia**
Idade avançada
Síndrome coronariana aguda
Infarto agudo do miocárdio prévio
Doença coronariana extensa/complexa
Diabetes melito
Doença renal crônica

*DAPT: dupla antiagregação plaquetária.*

Reconhece-se que, no perioperatório, ocorre aumento da trombogenicidade, resultante da agressão cirúrgica e da resposta inflamatória desencadeada por fatores como neoplasia, infecção, traumatismo ou isquemia. A maior trombogenicidade já é identificada como fator de risco para complicações cardiovasculares após operações não cardíacas,^13^ e a interrupção precoce da terapia antiplaquetária potencializa ainda mais o risco dessas complicações.^14^ **Dessa forma, intervenções cirúrgicas completamente eletivas devem ser realizadas após o término do tempo ideal de DAPT.** Entretanto, algumas situações, mesmo sem configurar urgência, requerem individualização de conduta por serem tempo-sensíveis: o adiamento a médio prazo compromete o prognóstico da doença de base. Essa é a situação da maioria das indicações de cirurgia oncológica.

Registros prospectivos apontam que entre 4,4% e 11% dos pacientes submetidos à ICP necessitam de cirurgia não cardíaca ao longo do primeiro ano.^15 , 16^ Dados recentes de grande registro italiano, com seguimento prospectivo de 39.362 pacientes pós-ICP, evidenciaram que, já nos primeiros 6 meses, 5,1% dos pacientes necessitaram de cirurgia não cardíaca, e 9,1% deles tinham sido operados ao final do primeiro ano.^17^

A recomendação da DAPT, com associação de ácido acetilsalicílico e um inibidor de P2Y12 (clopidogrel, ticagrelor ou prasugrel) após ICP, varia conforme o contexto clínico (doença arterial aguda ou crônica) e tipo de *stent* utilizado. Em pacientes submetidos à ICP na vigência de síndrome coronariana aguda (SCA), recomenda-se a manutenção por 12 meses, podendo a terapia ser reduzida para 6 meses ou estendida, levando-se em consideração o risco individual de sangramento, bem como o risco isquêmico.^18 - 21^ Já no contexto de doença arterial coronariana (DAC) crônica, a recomendação atual é a realização de DAPT durante tempo mínimo de 4 a 6 semanas para *stents* não farmacológicos, e geralmente de 3 a 12 meses para os *stents* farmacológicos, com abreviação excepcionalmente em 30 dias, a depender do risco de sangramento e geração do *stent* implantado.^18 - 21^

Fora do contexto perioperatório, muito se discute acerca da extensão da terapia antitrombótica, notadamente após SCA, para redução de eventos isquêmicos.^22^ Contudo, na interface perioperatória, o mais comum é termos que lidar com o intervalo mínimo de segurança para interromper a DAPT, ainda que transitoriamente, para, posteriormente, reiniciá-la.

Série emblemática de 40 pacientes submetidos à cirurgia não cardíaca nas primeiras 6 semanas após ICP com *stent* convencional trouxe atenção direcionada ao tópico, em virtude de seus resultados catastróficos: 20% de mortalidade,17,5% de infarto agudo do miocárdio (IAM) e 27,5% de sangramento grave no perioperatório.^23^ Apesar de outras séries retrospectivas que analisaram complicações cardiovasculares perioperatórias em pacientes com ICP e *stent* não farmacológico prévio^24 - 31^ terem mostrado menor incidência absoluta de eventos, reforçaram o conceito do impacto do intervalo entre os procedimentos no prognóstico, e o intervalo mínimo de segurança variou de 4^29 - 31^ a 6 semanas.^24 - 28^ Alguns anos depois, o uso dos *stents* farmacológicos de primeira geração foi acompanhado por maiores taxas de trombose tardia, gerando insegurança sobre o tempo ideal de DAPT, sendo estabelecida a duração mínima de 1 ano. Para pacientes submetidos à cirurgia não cardíaca no primeiro ano após ICP com *stent* farmacológico de primeira geração, a primeira semana após a operação conferiu risco 27 vezes maior de óbito ou IAM que as demais semanas.^15^ Nessa época, uma força-tarefa das Sociedades de Cardiologia Norte-Americanas e do Colégio Americano de Cirurgiões estabeleceu que toda cirurgia não cardíaca eletiva deveria ser adiada até completar pelo menos 1 ano de terapia, e pacientes com perspectiva de necessitarem de operação em curto prazo não deveriam realizar angioplastia com *stent* farmacológico.^32^ Outras séries retrospectivas daquela mesma época já sugeriam que os 6 primeiros meses eram os mais críticos.^27 , 28 , 31^ Esses resultados marcantes ainda embasam muitas das recomendações quanto ao manejo perioperatório de pacientes com angioplastia coronária nos dias atuais.

Em 2016, Holcomb et al.^33^ publicaram interessante análise retrospectiva de 9.381 procedimentos cirúrgicos não cardíacos, realizados, em média, 332 dias após ICP. Concluíram que a incidência de eventos cardíacos adversos após cirurgia não cardíaca era maior nos pacientes com *stent* coronariano em comparação à população pareada por características clínicas e que não necessitou de angioplastia com *stent* (5,7% *vs.* 3,6%; p < 0,001). Por outro lado, o tipo de *stent* – farmacológico ou não – não foi uma característica determinante significativa para o prognóstico (6% *vs.* 5,3%, p = 0,30, respectivamente).^33^ Por fim, o mesmo grupo demonstrou que o contexto clínico da ICP influenciou o efeito do intervalo entre os procedimentos para a ocorrência de eventos cardíacos perioperatórios. Assim, para os pacientes que realizaram ICP no contexto de IAM, o risco de complicação perioperatória era expressivamente maior nos primeiros 3 a 6 meses, sugerindo que o contexto clínico era mais relevante que a plataforma do *stent* utilizada,^34^ mostrando a necessidade de mudança no paradigma anterior, que indicava preferência pelo *stent* não farmacológico na condição de uma perspectiva de cirurgia não cardíaca no curto ou médio prazo.

Em recente estudo prospectivo e randomizado, a utilização de *stent* farmacológico (recoberto com biolimus A9, sem polímero) em associação ao uso de DAPT por 1 mês apresentou resultados melhores que *stent* não farmacológico no que diz respeito à incidência de eventos cardíacos adversos. Entre os 2.466 pacientes estudados, 278 foram operados no primeiro ano após a ICP, e a análise desse subgrupo replicou os dados globais, evidenciando menor necessidade de revascularização da lesão-alvo nos pacientes com *stent* farmacológico (HR 0,28, IC 0,08-0,99, p = 0,04). A incidência de óbito cardíaco, IAM ou trombose de *stent* foi 4,7% em 1 ano entre os pacientes com *stent* farmacológico e 10,1% para os pacientes com *stent* não farmacológico (HR 0,46, IC 0,18-1,18; p = 0,09). Um achado adicional foi que o menor intervalo entre a cirurgia e a ICP tinha impacto na incidência de evento cardíaco adverso entre os pacientes que receberam *stent* convencional: 14,9% para intervalo < 3 meses *vs.* 4,4% para intervalo de 4 a 12 meses; HR 3.586 (IC 1.012-12.709, p = 0,03).^35^ O intervalo não impactou o prognóstico dos pacientes que receberam *stent* farmacológico: 4,69% *vs.* 4,66%, HR 1.056 (IC 0.213-5.232, p = 0.947).^36^ Cabe ressaltar que os procedimentos cirúrgicos ocorreram após o término dos 30 dias de DAPT.

**A previsão de cirurgia não cardíaca não deve motivar a preferência por *stent* não farmacológico, conceito antigo e que não parece mais adequado frente às novas evidências.**

A seguir, pretendemos discutir as mais novas evidências sobre encurtamento da duração de DAPT ( Tabela 3 ) e, finalmente, contextualizá-las para o perioperatório, com proposta ainda não incorporada nas últimas versões da Diretriz de Avaliação Perioperatória da Sociedade Brasileira de Cardiologia.^19^

**Tabela 3:** – Resumo dos principais estudos que testaram DAPT curta (3 meses e 1 mês) em comparação a 12 meses após angioplastia com stent farmacológico

	Tempo de DAPT	N	Inibidor de P2Y12	AntiagregaNte mantido após tÉrmino da DAPT	N DAC/sca	Stent	Resultado
**OPTIMIZE, 2013**	3 vs. 12 meses	3.119	Clopidogrel	Ácido acetilsalicílico	DAC: 2.123 SCA: 996 IAMSST:168	Farmacológico eluído com zotarolimus (Medtronic Inc)	Não inferioridade do regime de 3 meses para morte por todas as causas, IAM, AVC ou sangramentos maiores.
**SMART CHOICE, 2019**	3 vs. 12 meses	2.912	Clopidogrel, ticagrelor ou prasugrel	Inibidor P2Y12 (75% clopidogrel)	DAC:1.250 AI: 958 IAMSST: 469 IAMCSST: 314	Farmacológico eluído com everolimus (Xience Prime/ Expedition/Alpine) ou sirolimus (Promus Element/ Premier, SYNERGY)	Não inferioridade do regime de 3 meses para mortalidade por todas as causas, AVC e IAM. Redução de sangramentos BARC 2-5.
**TWILIGHT, 2019**	3 vs. 12 meses	7.119	Ticagrelor	Ticagrelor	DAC: 2.503 AI: 2.494 IAMSST: 2.120	Farmacológico	Não inferioridade do regime de 3 meses para mortalidade por todas as causas, AVC e IAM. Redução de sangramentos BARC 2-5.
**TICO, 2020**	3 vs. 12 meses	3.056	Ticagrelor	Ticagrelor	AI: 926 IAMSST: 1.027 IAMCSST: 1.103	Farmacológico eluído com sirolimus (Orsiro, Biotronik)	Não inferioridade do regime de 3 meses para eventos cardiovasculares. Redução de sangramentos maiores e menores (TIMI).
**GLOBAL LEADERS, 2018**	1 vs. 12 meses	15 968	Ticagrelor	Ticagrelor	DAC: 8.481 AI: 2.022 IAMSST: 3.373 IAMCSST: 2.092	Farmacológico eluído com biolimus	Sem diferenças estatisticamente significantes em relação à incidência de morte por todas as causas, IAM e sangramento.
**STOPDAPT-2, 2019**	1 vs. 12 meses	3.045	Clopidogrel ou prasugrel	Clopidogrel	DAC: 1.861 AI: 407 IAMSST: 180 IAMCSST: 561	Farmacológico de cromo-cobalto eluído com everolimus (Xience)	Não inferioridade do regime de 1 mês para morte cardiovascular, IAM, AVC, trombose de stent e sangramentos maiores ou menores.
**LEADERS-FREE, 2015**	DAPT 1 mês; stent eluído com biolimus-A9 sem polímero vs bare-metal	2.466	Clopidogrel	Escolha do médico assistente Ácido acetilsalicílico em 85%	DAC: 1.403 AI: 370 IAMSST: 554 IAMCSST: 105	Farmacológico eluído com biolimus A9 sem polímero (BioFreedom) e bare-metal	Menor incidência de morte cardiovascular, IAM e trombose de stent no grupo com stent farmacológico.
**ONYX ONE, 2020**	DAPT 1 mês; Stent eluído com zotarolimus sem polímero vs stent eluído com biolimus-A9 sem polímero	1.996	Clopidogrel, ticagrelor ou prasugrel	Escolha do médico assistente Ácido acetilsalicílico em 56,2%	DAC: 729 AI: 360 IAMSST: 514 IAMCSTT: 108	Farmacológico eluído com zotarolimus com polímero (Medtronic) e stent eluído com biolimus-A9 sem polímero (BioFreedom)	Não inferioridade do stent farmacológico com polímero em relação à incidência de morte cardiovascular, IAM e trombose de stent.

*DAC: doença coronariana crônica; SCA: síndrome coronariana aguda; AI: angina instável; IAMSST: infarto agudo do miocárdio sem supradesnivelamento do seguimento ST; IAMCSST: infarto agudo do miocárdio com supradesnivelamento do seguimento ST; AVC: acidente vascular cerebral.*

## 2. Redução do Tempo de DAPT

Entre os estudos que testaram 3 meses de DAPT, destaca-se o **OPTIMIZE** ( *Optimized Duration of Clopidogrel Therapy Following Treatment with the Zotarolimus-Eluting Stent in Real-World Clinical Practice* ), que avaliou a não inferioridade de 3 meses de DAPT após ICP com *stent* farmacológico de segunda geração em relação a 12 meses, e a continuidade da monoterapia antiplaquetária era feita com ácido acetilsalicílico. Esse estudo envolveu 3.119 pacientes com DAC crônica ou SCA de baixo risco (angina instável ou IAM sem supra ST, após retorno da troponina para nível normal) em 33 centros brasileiros. Não houve diferenças significativas no que diz respeito à morte por todas as causas, IAM, acidente vascular encefálico (AVE) ou sangramentos maiores (6,0% *vs.* 5,8%, p = 0,002 para não inferioridade).^37^ Em consonância, o **SMART CHOICE** ( *Smart Angioplasty Research Team: Comparison Between P2Y12 Antagonist Monotherapy vs Dual Antiplatelet Therapy in Patients Undergoing Implantation of Coronary Drug-Eluting Stents* ), conduzido na Coreia, também demonstrou resultados favoráveis na utilização de DAPT por apenas 3 meses, com taxas de mortalidade, AVE e IAM não inferiores ao grupo que completou 1 ano de DAPT: 2,9% *vs.* 2,5%; p = 0,007 (para não inferioridade). Diferentemente do estudo OPTIMIZE, a monoterapia era mantida com o inibidor de P2Y12 (clopidogrel em mais de 75% dos casos) e, nesse estudo, mais da metade dos pacientes realizou ICP no contexto agudo, incluindo 10% de pacientes com IAM com supra do segmento ST.^38^

O estudo TWILIGHT *(Ticagrelor with Aspirin or Alone in High-Risk Patients after Coronary Intervention* ) comparou desfechos hemorrágicos e isquêmicos em pacientes de alto risco isquêmico ou hemorrágico submetidos a ICP com *stent* farmacológico, randomizados para DAPT com ácido acetilsalicílico e ticagrelor por 1 ano ou DAPT por 3 meses seguido de monoterapia com ticagrelor. Entre os 7.119 pacientes estudados, 64,8% realizaram ICP no contexto de angina instável ou IAM sem supra do segmento ST e houve redução de 44% da taxa de sangramento clinicamente relevante no grupo com encurtamento da DAPT (4% *vs* . 7,1%, p < 0,001); não houve diferença na incidência de IAM, AVE ou morte entre os grupos (3,9% em ambos os grupos, p < 0,001 para não inferioridade).^39^

No mesmo sentido, recentemente, foram publicados os resultados parciais do estudo EVOLVE short DAPT ( *Evaluation of 3-Month Dual Antiplatelet Therapy in High Bleeding Risk Patients Treated with a Bioabsorbable Polymer-Coated Everolimus-Eluting Stent* ), que avaliou a segurança da redução da DAPT, também para 3 meses, em pacientes com alto risco de sangramento e uso de *stent* farmacológico eluído com everolimus e com polímero bioabsorvível (Synergy). Depois de 15 meses de seguimento dos 1.487 pacientes que mantiveram apenas ácido acetilsalicílico após os 3 meses de DAPT, foi verificada a não inferioridade do uso de DAPT por 3 meses em relação à incidência de morte e IAM (5,6% *vs* . 5,7%, p = 0,0016 para não inferioridade), e a taxa de trombose de *stent* foi de apenas 0,2%. Ressalta-se que pacientes com IAM ou lesões complexas não foram incluídos nesse estudo.^40^

Finalmente, a estratégia de encurtar DAPT foi testada em pacientes com SCA, incluindo IAM com supra de ST (36% da população), no estudo TICO ( *Effect of Ticagrelor Monotherapy vs. Ticagrelor with Aspirin on Major Bleeding and Cardiovascular Events in Patients With Acute Coronary Syndrome* ). Nesse estudo, realizado em 38 centros da Coreia do Sul, em 3.056 indivíduos submetidos à ICP com *stent* farmacológico eluídos com sirolimus (ORSIRO), foi comparado o uso de ácido acetilsalicílico + ticagrelor por 3 meses, seguido de monoterapia com ticagrelor, *vs.* 12 meses de ácido acetilsalicílico e ticagrelor. Foi observada redução de 34% no desfecho primário composto por sangramentos maiores e eventos cardiovasculares no grupo que utilizou DAPT por apenas 3 meses (3,9% *vs* . 5,9%, HR 0,66, IC 0,48-0,92; p = 0,01). A diferença ocorreu em virtude da menor incidência das complicações hemorrágicas, sem diferença significativa na incidência de eventos cardiovasculares.^41^

A estratégia de duração de DAPT ainda mais curta, de apenas 1 mês após ICP com *stent* farmacológico, também apresenta evidências favoráveis em estudos que incluíram proporções semelhantes de pacientes no contexto eletivo e agudo. O estudo LEADERS FREE ( *Polymer-free Drug-Coated Coronary Stents in Patients at High Bleeding Risk* ) foi conduzido para comparar os resultados de angioplastia com *stent* farmacológico (recoberto com umirolimus, sem polímero) e *stent* não farmacológico, em população de alto risco de sangramento e previsão de apenas 1 mês de DAPT. Entre os 2.466 pacientes randomizados e seguidos durante 390 dias, o *stent* farmacológico foi superior em relação ao desfecho primário de eficácia, com menor necessidade de revascularização da lesão alvo (5,1% *vs* . 9,8%; p < 0,001), bem como em relação ao desfecho primário de segurança: 9,4% de óbito de causa cardiovascular, IAM ou trombose de *stent* em 390 dias *vs.* 12,9%, HR 0,71; IC 0,56-0,91, p = 0,005. Ressalta-se que, nesse estudo, 16% dos pacientes foram incluídos por terem cirurgia não cardíaca de grande porte planejada para o próximo ano, e, conforme previamente descrito, a segurança da estratégia se confirmou na população efetivamente operada.^35 , 36^

O estudo ONYX ONE ( *Polymer-based or Polymer-free Stents in Patients at High Bleeding Risk* ) demonstrou a não inferioridade do *stent* farmacológico com zotarolimus e polímero permanente (Resolute Onyx, Medtronic^TM^) em relação ao *stent* farmacológico recoberto com umirolimus e sem polímero, após 1 mês de DAPT seguida de terapia antiplaquetária única, no que diz respeito ao desfecho primário composto de IAM, trombose de *stent* ou morte cardiovascular (17,1% *vs.* 16,9%, p = 0,01 para não inferioridade).^42^ Nesse estudo, o *stent* farmacológico sem polímero, avaliado no estudo LEADERS FREE, foi assumido como comparador de menor trombogenicidade, e não mais o *stent* não farmacológico.^35^ Outro importante estudo que avaliou o encurtamento de DAPT foi o STOPDAPT-2 *(Short and Optimal Duration os Dual Antiplatelet Therapy After Everolimus-Eluting Cobalt-Chromium Stent-2* ), que envolveu pacientes com DAC estável e SCA e comparou a eficácia de DAPT por 1 *vs.* 12 meses após implante de *stent* farmacológico de segunda geração com everolimus e polímero permanente (Xience), demonstrando a não inferioridade da terapia farmacológica por 30 dias em relação ao desfecho composto primário de morte cardiovascular, IAM, AVE, trombose de *stent* , sangramentos maiores e menores.^43^ Já o GLOBAL LEADERS ( *Ticagrelor plus aspirin for 1 month, followed by ticagrelor monotherapy for 23 months vs aspirin plus clopidogrel or ticagrelor for 12 months, followed by aspirin monotherapy for 12 months after implantation of a drug-eluting stent: a multicentre, open-label, randomised superiority trial* ) foi um estudo que tentou demonstrar superioridade da estratégia de ticagrelor e ácido acetilsalicílico por 1 mês, seguida de ticagrelor por 23 meses, em relação a DAPT por 12 meses, seguida de ácido acetilsalicílico por mais 12 meses. Não foi evidenciada diferença significativa na mortalidade global ou incidência de IAM até 2 anos de seguimento entre os grupos estudados.^44^

Recentemente, os dados do estudo XIENCE Short-DAPT foram apresentados, e demonstrou-se não inferioridade em relação a mortalidade e IAM entre as estratégias de DAPT de curta duração (1 ou 3 meses) ou longa duração (por até 12 meses); todos os pacientes foram submetidos à angioplastia eletiva com o *stent* XIENCE, e eram de alto risco para sangramento. O ácido acetilsalicílico foi mantido como monoterapia após a interrupção da DAPT, e, embora 34% dos pacientes tenham sido submetidos à ICP para tratamento de SCA, aqueles com infarto com supra do segmento ST foram excluídos, além dos pacientes com lesão de tronco de coronária esquerda, lesão de enxerto, lesão com trombo, ou até mesmo tratamento de reestenose de *stent* . Também foram excluídos pacientes com programação de cirurgia durante o tempo mínimo planejado de DAPT (1 ou 3 meses).^45^

Recente metanálise envolvendo 79.073 pacientes analisou a utilização dos antiplaquetários (quatro grupos de DAPT), categorizados pela duração, no que diz respeito a eventos isquêmicos e hemorrágicos após ICP com *stent* farmacológico. A referência para comparação foi a DAPT convencional (12 meses), e os outros grupos foram DAPT estendida (> 12 meses), DAPT média (6 meses) e DAPT curta (< 6 meses). De maneira geral, não foi observada diferença na mortalidade entre as diferentes durações de DAPT. A extensão da DAPT por mais de 1 ano propiciou redução de IAM, porém, sem benefício líquido favorável, exceto para pacientes com SCA e baixo risco de sangramento. Por outro lado, a redução da duração da DAPT para 1 ou 3 meses foi não inferior à duração de 12 meses, ou mesmo de 6 meses, no que diz respeito a eventos isquêmicos. A manutenção de monoterapia com inibidor de P2Y12 após curto período de DAPT conferiu o melhor benefício líquido, com redução de sangramentos.^46^

**Figura 1:**
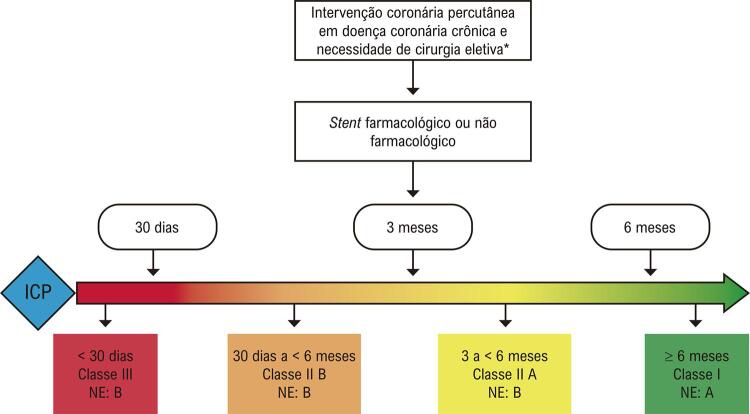
– Fluxograma para definição do intervalo entre cirurgia não cardíaca e ICP para pacientes submetidos à ICP eletiva.

O conjunto das evidências expostas embasam certa segurança para que, no contexto de proposta de cirurgia não cardíaca tempo-sensível, em que se configura alto risco hemorrágico, a DAPT seja abreviada para 3 meses ou até mesmo 1 mês. O que, até há pouco tempo, se preconizava como ideal aguardar 6 meses entre ICP eletiva com *stent* farmacológico e cirurgia não cardíaca, hoje, seguramente, podemos considerar 3 meses para os *stents* de nova geração. Em situações mais prementes, já há evidências para interrupção de DAPT em 30 dias, analogamente ao que se faz nesse contexto para pacientes com *stent* não farmacológico. Antes desse período, apenas operações não cardíacas de urgência ou emergência se justificam ( Figura 1 ).

* Intervenções cirúrgicas não cardíacas completamente eletivas devem ser realizadas após o tempo ideal de dupla antiagregação plaquetária

**Figura 2:**
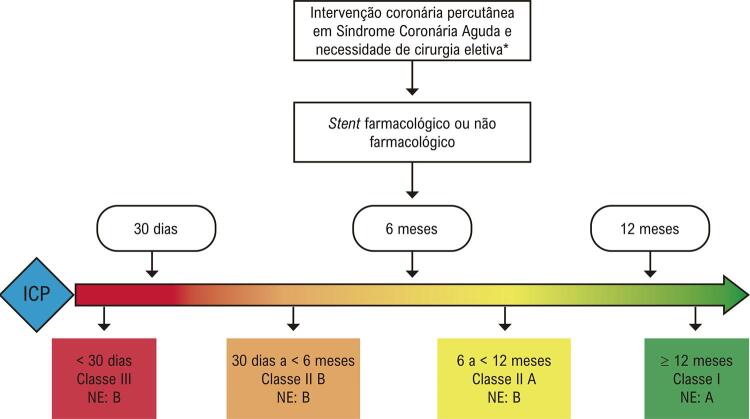
– Fluxograma para definição do intervalo entre cirurgia não cardíaca e ICP para pacientes submetidos à ICP no contexto de síndrome coronariana aguda.

Para pacientes que realizaram ICP no contexto agudo, o ideal é completar 1 ano de DAPT antes de operações eletivas; entretanto, na necessidade de procedimentos prementes, o intervalo pode ser reduzido para 6 meses e, excepcionalmente, 1 mês ( Figura 2 ).

* Intervenções cirúrgicas não cardíacas completamente eletivas devem ser realizadas após o tempo ideal de dupla antiagregação plaquetária

Devido à complexidade do manejo da terapia antitrombótica no perioperatório de cirurgias não cardíacas, a decisão sobre o encurtamento de DAPT deve levar em conta o risco individual e cirúrgico de complicações trombóticas e hemorrágicas, sendo idealmente compartilhada entre o cardiologista clínico, o cardiologista intervencionista e a equipe cirúrgica.

### 2.1. Recomendações sobre Intervalo entre Cirurgia Não Cardíaca Eletiva e Intervenção Coronária Percutânea

## 3. Cuidado Perioperatório Imediato

### 3.1. Manter um dos Antiagregantes

Em todas essas situações, apenas um antiagregante deve ser retirado, visto que a retirada de ambos está relacionada a menor intervalo entre a modificação da DAPT e trombose de *stent* ^47^ – exceção para procedimentos de altíssimo risco hemorrágico, notadamente **neurocirurgias** , em que ambos antiagregantes devem ser suspensos e reiniciados o mais precocemente possível. A recomendação consiste em manutenção do ácido acetilsalicílico e retirada do inibidor de P2Y12 alguns dias antes do procedimento, a depender do fármaco utilizado. No caso de uso de dupla antiagregação com clopidogrel, deve-se suspender seu uso 5 dias antes do procedimento, com reintrodução após certificação de boa hemostasia, em acordo com a equipe cirúrgica. Idealmente, a DAPT não deve ser suspensa por período superior a 10 dias no perioperatório.^47^ Quando em uso do ticagrelor, a recomendação ainda permanece de suspensão do fármaco 5 dias antes do procedimento, apesar do já conhecido perfil farmacocinético de mais rápida recuperação da atividade plaquetária quando comparado ao uso de clopidogrel.^7^ Tal fato é corroborado pelos resultados de subanálise dos pacientes submetidos à revascularização cirúrgica do miocárdio no estudo PLATO ( *Platelet inhibition and patients Outcomes* ), em que foram analisados indivíduos randomizados para ácido acetilsalicílico associado a clopidogrel ou ticagrelor, demonstrando menor taxa de sangramentos no grupo em utilização da nova geração de inibidores de P2Y12.^48^ Já o prasugrel deve ser suspenso 7 dias antes de cirurgias não cardíacas.^49^

Com o intuito de avaliar a possibilidade de redução do tempo de suspensão dos inibidores de P2Y12 no contexto pré-operatório, foi realizado o estudo PLAT-CABG ( *Platelet Reactivity in Patients with Acute Coronary Syndromes Awaiting Surgical Revascularization* ), que utilizou teste de reatividade plaquetária antes de cirurgias de revascularização miocárdica em pacientes recebendo ácido acetilsalicílico e clopidogrel, no contexto de SCA. Nesse estudo brasileiro, unicêntrico, de não inferioridade, foi comparado o tempo de suspensão usual do clopidogrel de 5 dias antes da cirurgia com a utilização do teste de reatividade plaquetária a adenosina difosfato para guiar o momento da cirurgia. Foi verificada, no grupo de intervenção, redução do tempo para realização do procedimento cirúrgico (112 horas *vs* . 136 horas, p < 0,001), sem impacto em relação a eventos hemorrágicos maiores no pós-operatório.^50^ Ainda não há recomendações específicas para o uso assistencial sistemático dos testes de agregabilidade plaquetária. Por outro lado, para situações mais prementes, em que não há emergência cirúrgica, mas com interesse clínico (com base em avaliação individual) em encurtar a espera em 1 ou 2 dias, pode ser atrativo considerar tal prática desde que em comum acordo com a equipe cirúrgica.

O conceito de “terapia de ponte”, arraigado no cuidado de pacientes que fazem uso de anticoagulação oral com varfarina, é lembrado no perioperatório de pacientes com DAPT. Entretanto, alguns pontos merecem ressalva. A utilização de heparina de baixo peso molecular não substitui efetivamente a terapia antiagregante, e pode até mesmo aumentar a incidência de eventos hemorrágicos e trombóticos.^51^ Por outro lado, a utilização de antiagregação parenteral, com meia-vida mais curta (inibidores de glicoproteína IIb, IIIa), foi testada em séries de casos,^52 , 53^ constituindo indicação classe IIb pelas diretrizes atuais, naquelas situações de risco trombótico muito elevado. Sugerimos considerar essa possibilidade apenas para casos muito selecionados, notadamente quando a DAPT é interrompida com menos de 1 mês, após ICP complicada e em contexto agudo ( Tabela 4 ).

**Tabela 4:** – Recomendações para o manejo dos antiagregantes plaquetários no perioperatório

Sumário de recomendações	Classe de recomendação	Nível de evidência
Manter o ácido acetilsalicílico na dose de 100mg ao dia durante todo o perioperatório, exceto para neurocirurgias ou procedimentos de risco hemorrágico proibitivo.	I	A
Suspender clopidogrel e ticagrelor 5 dias antes de cirurgias não cardíacas.	I	B
Suspender prasugrel 7 dias antes de cirurgias não cardíacas.	I	B
Em caso de interrumpção de DAPT antes do tempo mínimo ideal, realizar cirurgias não cardíacas em centros com suporte multidisciplinar e retaguarda hemodinâmica.	I	C
Realizar cirurgias de baixo risco de sangramento na vigência de DAPT se o intervalo decorrido desde a angioplastia for menor do que 3 meses.	IIa	C
Utilizar teste de agregabilidade plaquetária para abreviar tempo de suspensão de inibidor de P2Y antes de cirurgias não cardíacas.	IIb	B
Para casos de risco trombótico muito elevado (menos de 1 mês de ICP e interrupção de DAPT, utilizar tirofiban como terapia de ponte.	IIb	B
Realizar terapia de ponte com heparina de baixo peso molecular.	III	B

Conforme descrito, o conhecimento e a tecnologia caminham no sentido de encurtar o tempo de DAPT, beneficiando, sobretudo, pacientes de alto risco de sangramento. Contudo, sempre que a DAPT for interrompida antes do planejado, é de fundamental importância que a cirurgia seja realizada em centros com suporte multidisciplinar, para monitoramento cardiovascular e retaguarda hemodinâmica, em caso de complicação.

## 4. Revascularização Miocárdica Profilática

A discussão a respeito do intervalo ideal entre intervenção coronária percutânea e cirurgia não cardíaca pode ocorrer pela necessidade de cirurgia em paciente com ICP recente ou, eventualmente, no planejamento de revascularização miocárdica durante a avaliação pré-operatória do risco cardíaco. Os estudos randomizados que analisaram o impacto da revascularização miocárdica profilática na redução de eventos isquêmicos em pacientes com planejamento de cirurgia vascular não demonstraram impacto significativo da estratégia.^54 - 56^ Dessa maneira, a revascularização miocárdica é recomendada apenas em indivíduos com indicação inequívoca ao procedimento, independentemente do contexto perioperatório, e não deve ser realizada de rotina com objetivo exclusivo de reduzir complicações cardiovasculares no perioperatório. Nesses casos, para tomada de decisão, sempre deve-se levar em consideração o contexto clínico do paciente, prognóstico da doença base que levou à indicação do procedimento cirúrgico, assim como a necessidade de respeitar o intervalo mínimo de DAPT para as intervenções coronárias percutâneas e o risco de sangramento associado à intervenção. Cabe ressaltar o que previamente já foi exposto, que a previsão de cirurgia não cardíaca não deve motivar a preferência por *stent* não farmacológico, conceito antigo e que não parece mais adequado frente às novas evidências. Outra questão a ser lembrada nas situações de indicação de ICP por risco isquêmico muito elevado e necessidade de operação não cardíaca tempo-sensível é a possibilidade de ICP exclusivamente com balão, sem *stent* . Há pouca evidência sobre a estratégia, com uso apenas de ácido acetilsalicílico e intervalo de pelo menos 2 semanas até a operação.^57^ Não há garantia na fase de planejamento de que isso seja viável, pois o *stent* pode ser necessário para assegurar o resultado primário da intervenção, de forma que essa estratégia não deve ser utilizada rotineiramente.

As indicações específicas de revascularização miocárdica devem seguir as recomendações das diretrizes de doença coronariana crônica e aguda.^58 , 59^
