# Supplementary material for: Atualização da Diretriz de Avaliação Cardiovascular Perioperatória da Sociedade Brasileira de Cardiologia: Foco em Manejo dos Pacientes com Intervenção Coronária Percutânea – 2022
Source: Arq Bras Cardiol. 2022 Feb 14;118(2):536–47. [Article in Portuguese] doi: 10.36660/abc.20220039 (PMC8856674; doi:10.36660/abc.20220039)
Supplement: Translation

# Translation: Update of the Brazilian Society of Cardiology’s Perioperative Cardiovascular Assessment Guideline: Focus on Managing Patients with Percutaneous Coronary Intervention – 2022

**Authors:** Daniela Calderaro, Luciana Dornfeld Bichuette, Pamela Camara Maciel, Francisco Akira Malta Cardozo, Henrique Barbosa Ribeiro, Danielle Menosi Gualandro, Luciano Moreira Baracioli, Alexandre de Matos Soeiro, Carlos Vicente Serrano, Ricardo Alves da Costa, Bruno Caramelli

**Affiliations:** 1 Brazil Instituto do Coração, Hospital das Clínicas HCFMUSP, Faculdade de Medicina, Universidade de São Paulo, São Paulo, SP- Brazil; 2 Switzerland Department of Cardiology and Cardiovascular Research Institute Basel, University Hospital Basel, University of Basel, Basel – Switzerland; 3 Brazil Syrian Lebanese Hospital, São Paulo, SP – Brazil; 4 Brazil Mirante Portuguese Charity Hospital, São Paulo, SP – Brazil; 5 Brazil Dante Pazzanese Institute of Cardiology, São Paulo, SP – Brazil

**Table:** 

Update of the Brazilian Society of Cardiology’s Perioperative Cardiovascular Assessment Guideline: Focus on Managing Patients with Percutaneous Coronary Intervention – 2022	
The report below lists declarations of interest as reported to the SBC by the experts during the period of the development of these statement, 2021.	
Expert	Type of relationship with industry
Alexandre de Matos Soeiro	Funding of continuing medical education activities, including travel, accommodation and registration in conferences and courses, from the brazilian or international pharmaceutical, orthosis, prosthesis, equipment and implants industry: - Bayer, Pfizer, Daiichi Sankyo, Biolab.
Bruno Caramelli	Nothing to be declared
Carlos Vicente Serrano Jr.	Nothing to be declared
Daniela Calderaro	Financial declaration A - Economically relevant payments of any kind made to (i) you, (ii) your spouse/partner or any other person living with you, (iii) any legal person in which any of these is either a direct or indirect controlling owner, business partner, shareholder or participant; any payments received for lectures, lessons, training instruction, compensation, fees paid for participation in advisory boards, investigative boards or other committees, etc. From the brazilian or international pharmaceutical, orthosis, prosthesis, equipment and implants industry: - Bayer: Xarelto and Finerinone; Janssen: pulmonary hypertension Other relationships Funding of continuing medical education activities, including travel, accommodation and registration in conferences and courses, from the brazilian or international pharmaceutical, orthosis, prosthesis, equipment and implants industry: - Bayer: Xarelto; Daiichi-Sankyo: Lixiana; Janssen: pulmonary hypertension
Danielle Menosi Gualandro	Nothing to be declared
Francisco Akira Malta Cardozo	Financial declaration A - Economically relevant payments of any kind made to (i) you, (ii) your spouse/partner or any other person living with you, (iii) any legal person in which any of these is either a direct or indirect controlling owner, business partner, shareholder or participant; any payments received for lectures, lessons, training instruction, compensation, fees paid for participation in advisory boards, investigative boards or other committees, etc. From the brazilian or international pharmaceutical, orthosis, prosthesis, equipment and implants industry: - Bayer: Xarelto. C - Personal research funding paid by the brazilian or international pharmaceutical, orthosis, prosthesis, equipment and implants industry: - Bayer: Xarelto. Other relationships Funding of continuing medical education activities, including travel, accommodation and registration in conferences and courses, from the brazilian or international pharmaceutical, orthosis, prosthesis, equipment and implants industry: - Bayer: Xarelto.
Henrique Barbosa Ribeiro	Nothing to be declared
Luciana Dornfeld Bichuette	Nothing to be declared
Luciano Moreira Baracioli	Other relationships Funding of continuing medical education activities, including travel, accommodation and registration in conferences and courses, from the brazilian or international pharmaceutical, orthosis, prosthesis, equipment and implants industry: - Bayer: Xarelto.
Pamela Camara Maciel	Nothing to be declared
Ricardo Alves da Costa	Nothing to be declared

## 1. Introduction

The care of patients undergoing percutaneous coronary intervention (PCI) and the eventual need for early non-cardiac surgery is one of the topics that generates the most debate in perioperative medicine, as it involves important questions about the management of antithrombotic therapy, in addition to the usual management issues of cardiac risk in coronary artery disease. On the one hand, the hemorrhagic risk inherent in surgery and potentiated by antiplatelet agents must be managed. On the other, the increased risk of stent thrombosis must also be considered, especially if dual antiplatelet therapy (DAPT) is shortened. Elements such as the urgency and extent of the surgical procedure, the patient’s clinical status, coronary angioplasty data (eg, the elapsed interval), an elective or emergency context, the primary results, and the stent type are essential for individualizing recommendations, considering both hemorrhagic risk ( Table 1 ) and thrombotic risk ( Table 2 ).

**Table 1:** – Factors associated with high risk of bleeding1-6

Factors associated with a high risk of bleeding
**1) Factors inherent to the procedure**
**Low risk:**
- Gastrointestinal procedures (eg, endoscopy, colonoscopy, and capsule endoscopy)
- Cardiovascular procedures (eg, pacemaker/ICD implantation or generator replacement, cardiac ablation, radial access coronary catheterization)
- Dermatological procedures (eg, skin biopsy)
- Ophthalmic procedures (eg, cataract surgery)
- Dental procedures (eg, extraction of up to two teeth and endodontic procedures)
**High risk:**
- Surgeries requiring neuraxial anesthesia
- Neurosurgeries
- Major vascular surgeries (eg, aortic aneurysm repair and carotid endarterectomy)
- Major abdominal surgeries (eg, neoplastic resection surgeries, resections for inflammatory bowel disease or diverticulitis)
- Major orthopedic surgeries (eg, hip and knee arthroplasty)
**2) Clinical factors**
History of bleeding
Use of oral anticoagulants
Female sex
Advanced age
Chronic kidney disease
Diabetes mellitus
Anemia
Thrombocytopenia
Chronic corticosteroid or non-steroidal anti-inflammatory drug use

*ICD: implantable cardioverter-defibrillator*

**Table 2:** – Risk factors associated with stent thrombosis and ischemic events after angioplasty7-12

Risk factors associated with stent thrombosis
Early interruption of DAPT
Acute coronary syndrome
Diabetes mellitus
Smoking
Neoplasm
Peripheral arterial disease
First-generation drug-eluting stent
Ventricular ejection fraction <40%
Proximal anterior descending coronary arterial lesion
Previous angioplasty
Coronary bifurcation stent
Small-diameter stent
In-stent restenosis
Undersized stent
Long stent
**Risk factors associated with ischemic events after angioplasty**
Advanced age
Acute Coronary Syndrome
Previous acute myocardial infarction
Extensive/complicated coronary heart disease
Diabetes mellitus
Chronic kidney disease

*DAPT: dual antiplatelet therapy*

It is recognized that, in the perioperative period, there is an increase in thrombogenicity, resulting from surgical aggression and the inflammatory response triggered by factors such as neoplasia, infection, trauma or ischemia. Greater thrombogenicity has already been identified as a risk factor for cardiovascular complications after noncardiac surgeries,^13^ and early interruption of antiplatelet therapy further increases the risk of these complications.^14^ **Thus, completely elective surgical interventions must be performed after the ideal course of DAPT has been completed.** However, some time-sensitive situations, even non-urgent ones, can require an individualized approach since medium-term delay can compromise prognosis. This is the case with most referrals for cancer surgery.

Prospective records show that between 4.4% and 11% of PCI patients require noncardiac surgery during the first year.^15 , 16^ Recent data from a large Italian registry with prospective follow-up of 39,362 post-PCI patients found that, in the first 6 months, 5.1% of the patients required noncardiac surgery and that 9.1% had undergone a surgery by the end of the first year.^17^

The recommendation for DAPT after PCI, an association of acetylsalicylic acid and a P2Y12 inhibitor (clopidogrel, ticagrelor or prasugrel), varies according to the clinical context (acute or chronic arterial disease) and stent type. In patients with acute coronary syndrome (ACS) who are undergoing PCI, 12 months of therapy is recommended, which can either be reduced to 6 months or extended based on the individual risk of bleeding and ischemic events.^18 - 21^ In the context of chronic coronary artery disease (CAD), the current recommendations for DAPT include a minimum of 4 to 6 weeks for bare-metal stents and 3 to 12 months for drug-eluting stents, which in certain cases may be abbreviated to 30 days, depending on the bleeding risk and stent type.^18 - 21^

Beyond the perioperative context, there is much debate about the length of antithrombotic therapy necessary to reduce ischemic events, especially in ACS.^22^ However, the most common issue in the perioperative period is how long DAPT can be safely interrupted before subsequent reinitialization.

A series of 40 patients who underwent noncardiac surgery in the first 6 weeks after PCI with bare-metal stents brought attention to the topic due to the catastrophic results: 20% mortality, 17.5% acute myocardial infarction (AMI) and 27.5% severe perioperative bleeding.^23^ Although other retrospective series on perioperative cardiovascular complications in PCI patients who received bare-metal stents^24 - 31^ have shown a lower absolute incidence of events, they reinforced the impact that the interval between procedures has on prognosis, with the minimum safe interval varying from 4^29 - 31^ to 6 weeks.^24 - 28^ A few years after these series, first-generation drug-eluting stents were introduced, which were accompanied by higher rates of late thrombosis. This led to uncertainty about the ideal duration of DAPT, resulting in the establishment of a 1-year minimum. In patients who underwent noncardiac surgery in the first year after PCI with a first-generation drug-eluting stent, there was a 27 times greater risk of death or AMI in the first week after the operation than in subsequent weeks.^15^ A task force from several organizations, including the American Heart Association and the American College of Surgeons, then advised that all elective noncardiac surgery should be postponed until at least 1 year of DAPT had been completed, and patients who required an operation in the short term should not undergo angioplasty with a drug-eluting stent.^32^ Other retrospective series from that period also suggested that the first 6 months were the most critical.^27 , 28 , 31^ These remarkable results still guide many recommendations about the perioperative management of coronary angioplasty patients.

In 2016, Holcomb et al.^33^ published an interesting retrospective analysis of 9,381 noncardiac surgical procedures that were performed an average of 332 days after PCI. They concluded that the incidence of adverse cardiac events after noncardiac surgery was higher in patients with coronary stents than in matched controls who did not require angioplasty with a stent (5.7% vs. 3.6%; p <0.001). On the other hand, the type of stent (drug-eluting or bare metal) did not significantly affect prognosis (6% vs. 5.3%, p = 0.30, respectively).^33^ Finally, the same group of authors demonstrated that the clinical context of PCI influenced the effect of the interval between procedures on the occurrence of perioperative cardiac events. Thus, for patients who underwent PCI in the context of AMI, the risk of perioperative complications was significantly higher in the first 3 to 6 months, which suggests that the clinical context was more relevant than stent type.^34^ This indicates that a change is needed in the previous paradigm, which preferred non-pharmacological stents for short- or medium-term noncardiac surgeries.

A recent prospective randomized study found that a drug-eluting stent (polymer-free, coated with BioLimus A9) in association with 1 month of DAPT resulted in fewer adverse cardiac events than bare-metal stents. Of the 2,466 patients, 278 underwent surgery in the first year after PCI, and this subgroup replicated the global data, showing that there is less need for target lesion revascularization with drug-eluting stents (hazard ratio [HR], 0.28; confidence interval [CI], 0.08-0.99; p = 0.04). The incidence of cardiac death, AMI, or stent thrombosis was 4.7% at 1 year among patients with drug-eluting stents and 10.1% for patients with bare-metal stents (HR, 0.46; CI, 0.18-1.18; p = 0.09). An additional finding was that the shorter interval between surgery and PCI affected the incidence of adverse cardiac events among patients who received bare-metal stents: 14.9% for an interval <3 months vs. 4.4% for an interval of 4-12 months (HR, 3.586; CI, 1.012-12,709; p = 0.03).^35^ The interval did not affect the prognosis of patients who received drug-eluting stents: 4.69% vs. 4.66% (HR, 1.056; CI, 0.213-5.232; p = 0.947).^36^ It is noteworthy that the surgical procedures took place after 30 days of DAPT.

**Predicted noncardiac surgery should not lead to a preference for non-pharmacological stents. This concept no longer seems valid in light of the new evidence.**

Next, we intend to discuss the newest evidence about shortening DAPT ( Table 3 ) and, finally, to contextualize it for the perioperative period, including a proposal not yet incorporated in the latest versions of the Brazilian Society of Cardiology’s Perioperative Assessment Guidelines.^19^

**Table 3:** – Summary of key studies testing 1 or 3 months vs 12 months of DAPT after drug-eluting stent angioplasty

	DAPT DURATION	N	P2Y12 INHIBITOR	ANTIPLATELET USED AFTER DAPT	N CAD/aCS	Stent TYPE	ResultS
**OPTIMIZE (2013)**	3 vs 12 months	3119	Clopidogrel	Acetylsalicylic acid	CAD: 2123 ACS: 996 STEMI: 168	Zotarolimus-eluting (Medtronic)	The 3-month regimen was not inferior for all-cause mortality, AMI, stroke, or major bleeding.
**SMART CHOICE (2019)**	3 vs 12 months	2912	Clopidogrel, ticagrelor or prasugrel	P2Y12 inhibitor (75% clopidogrel)	CAD: 1250 UA: 958 STEMI: 469 Non-STEMI: 314	Everolimus- (Xience Prime/Expedition/Alpine) or sirolimus- (Promus Element/Premier SYNERGY) -eluting	The 3-month regimen was not inferior for all-cause mortality, stroke, or AMI. BARC bleeding scale reduction of 2 to 5 points.
**TWILIGHT (2019)**	3 vs 12 months	7119	Ticagrelor	Ticagrelor	CAD: 2503 UA: 2494 STEMI: 2120	Drug-eluting	The 3-month regimen was not inferior for all-cause mortality, stroke, or AMI. BARC bleeding scale reduction of 2 to 5 points.
**TICO (2020)**	3 vs 12 months	3056	Ticagrelor	Ticagrelor	UA: 926 STEMI: 1027 Non-STEMI: 1103	Sirolimus-eluting (Orsiro Biotronik)	The 3-month regimen was not inferior for cardiovascular events. Reduction of major and minor bleeding (TIMI).
**GLOBAL LEADERS (2018)**	1 vs 12 months	15,968	Ticagrelor	Ticagrelor	CAD: 8481 UA: 2022 STEMI: 3373 Non-STEMI: 2092	BioLimus-eluting	No significant differences in all-cause mortality, AMI or bleeding.
**STOPDAPT-2 (2019)**	1 vs 12 months	3045	Clopidogrel or prasugrel	Clopidogrel	CAD: 1861 UA: 407 STEMI: 180 Non-STEMI: 561	Cobalt-chromium everolimus (Xience)- eluting	The 1-month regimen was not inferior for cardiovascular death, AMI, stroke, stent thrombosis, or major or minor bleeding.
**LEADERS FREE (2015)**	1 month	2466	Clopidogrel	Attending physician’s choice Acetylsalicylic acid (85%)	CAD: 1403 UA: 370 STEMI: 554 Non-STEMI: 105	Polymer-free BioLimus A9 (BioFreedom)-eluting vs bare-metal	Drug-eluting stents had a lower incidence of cardiovascular death, AMI, and stent thrombosis.
**ONYX ONE (2020)**	1 month	1996	Clopidogrel, ticagrelor, or prasugrel	Attending physician’s choice Acetylsalicylic acid (56.2%)	CAD: 729 UA: 360 STEMI: 514 Non-STEMI: 108	Polymer-based zotarolimus-eluting (Medtronic) and polymer-free BioLimus A9-eluting (BioFreedom)	The drug-eluting polymer stents were not inferior for cardiovascular death, AMI, or stent thrombosis.

*CAD: chronic coronary disease; ACS: acute coronary syndrome; UA: unstable angina; STEMI: acute myocardial infarction with ST segment elevation; non-STEMI: acute myocardial infarction without ST segment elevation .*

## 2. Early Interruption of Dual Antiplatelet Therapy

**The OPTIMIZE trial,** conducted at 33 Brazilian centers, tested 3 months vs 12 months of DAPT after PCI with second-generation drug-eluting stents, continuing antiplatelet monotherapy with acetylsalicylic acid. Its sample included 3,119 patients with chronic CAD or low-risk ACS (unstable angina or non-ST-elevation AMI after troponin returned to normal levels). No significant differences were found for all-cause mortality, AMI, stroke, or major bleeding (6.0% vs. 5.8%, p = 0.002 for non-inferiority).^37^ Accordingly, the **SMART CHOICE** trial, conducted in Korea, also found no significant differences between 3 months and 12 months of DAPT for mortality rates, stroke, or AMI: 2.9% vs. 2.5%; p = 0.007 (for non-inferiority). Unlike the OPTIMIZE trial, monotherapy was continued with a P2Y12 inhibitor (clopidogrel in more than 75% of cases) and more than half of the patients underwent acute PCI, including 10% with ST-segment elevation myocardial infarction (STEMI).^38^

The TWILIGHT study compared bleeding and ischemic outcomes in patients at high risk of ischemia or bleeding who underwent PCI with drug-eluting stents. The patients were randomized to DAPT with acetylsalicylic acid + ticagrelor for 1 year or DAPT for 3 months followed by ticagrelor monotherapy. Of the 7,119 participants, 64.8% underwent PCI in the context of unstable angina or non-STEMI. There was a 44% reduction in clinically relevant bleeding in the 3-month DAPT group (4% vs. 7.1 %, p <0.001). There was also no difference in the incidence of AMI, stroke or death between the groups (3.9% in both groups, p <0.001 for non-inferiority).^39^

The EVOLVE Short DAPT Study, whose partial results were recently published, evaluated the safety of reducing DAPT to 3 months in patients at high risk of bleeding, as well as the use of everolimus-eluting stents with a bioabsorbable polymer (Synergy). A total of 1,487 patients who received acetylsalicylic acid monotherapy after 3 months of DAPT were followed up for 15 months. It was confirmed that 3 months of DAPT was non-inferior to 12 months regarding mortality and AMI (5.6% vs. 5.7%, p = 0.0016 for non-inferiority), with a stent thrombosis rate of only 0.2%. It is noteworthy that patients with AMI or complex lesions were not included in this study.^40^

Finally, the TICO trial tested shortened DAPT in patients with ACS, including STEMI (36% of the population). This trial, which was conducted at 38 centers in South Korea, included 3,056 patients who underwent PCI with an Orsiro sirolimus-eluting stent, comparing acetylsalicylic acid + ticagrelor for 3 months followed by ticagrelor monotherapy with 12 months of acetylsalicylic acid + ticagrelor. A 34% reduction in the primary endpoint (major bleeding and cardiovascular events) was observed in the 3-month group (3.9% vs. 5.9%; HR, 0.66; CI 0.48-0.92; p = 0.01). The difference was due to the lower incidence of bleeding complications, with no significant difference in the incidence of cardiovascular events.^41^

Favorable evidence has also been found for even shorter DAPT duration, (ie, 1 month after PCI with drug-eluting stent) in studies with similar proportions of elective and acute patients. The LEADERS FREE trial was conducted to compare the results of angioplasty with either a polymer free umirolimus-eluting stent or a non-pharmacological stent in a population at high risk of bleeding, with only 1 month of DAPT predicted. In the 2,466 patients randomized and followed for 390 days, drug-eluting stents were superior for the primary efficacy endpoint (less need for target lesion revascularization: 5.1% vs. 9.8%; p <0.001) and the primary safety endpoint (death from cardiovascular causes, AMI, or stent thrombosis in 390 days: 9.4% vs. 12.9%; HR, 0.71; CI, 0.56-0.91; p = 0.005). It is noteworthy that 16% of the patients had major noncardiac surgery planned for the next year, and, as previously mentioned, the strategy’s safety was confirmed.^35 , 36^

The ONYX ONE trial demonstrated that polymer-based zotarolimus-eluting stents (Resolute Onyx, Medtronic) were not inferior to polymer-free umirolimus-eluting stents after 1 month of DAPT followed by antiplatelet monotherapy for the primary composite endpoint of AMI, stent thrombosis, or cardiovascular death (17.1% vs. 16.9%; p = 0.01 for non-inferiority).^42^ In ONYX ONE, the polymer-free drug-eluting stent tested in LEADERS FREE was assumed to be a comparator of lower thrombogenicity rather than a bare-metal stent.^35^ Another important study that evaluated shortened DAPT was the STOPDAPT-2 trial, which involved patients with stable CAD and ACS and compared the efficacy of 1 vs 12 months of DAPT after second-generation polymer-based (Xience) everolimus-eluting stent implantation. One month of DAPT was not inferior for the primary composite endpoint: cardiovascular death, AMI, stroke, stent thrombosis, and major and minor bleeding.^43^ GLOBAL LEADERS, a multicenter, open-label, randomized superiority trial, tested ticagrelor + aspirin for 1 month, followed by ticagrelor monotherapy for 23 months vs aspirin + clopidogrel or ticagrelor for 12 months, followed by aspirin monotherapy for 12 months, after implantation of a drug-eluting stent. The trial attempted to demonstrate the superiority of ticagrelor + acetylsalicylic acid for 1 month, followed by ticagrelor for 23 months, over 12 months of DAPT followed by acetylsalicylic acid for another 12 months. However, there was no significant difference in overall mortality or AMI incidence in up to 2 years of follow-up between the groups.^44^

Recently, data from the XIENCE Short-DAPT study demonstrated that short-term (1- or 3-month) DAPT strategies were not inferior to long-term (up to 12 months) strategies for mortality or AMI. All patients in this study underwent elective angioplasty with XIENCE stents and were at high risk for bleeding. Acetylsalicylic acid was continued as monotherapy after DAPT was interrupted and, although 34% of patients underwent PCI for ACS treatment, those with STEMI were excluded, in addition to patients with left coronary trunk lesion, graft injury, thrombus injury, or even in-stent restenosis treatment. Patients with a scheduled surgery during the minimum planned DAPT time (1 or 3 months) were also excluded.^45^

A recent meta-analysis of 79,073 patients analyzed the effects of four durations of DAPT on ischemic and hemorrhagic events after PCI with drug-eluting stents. The reference was conventional DAPT (12 months), which was compared with extended DAPT (>12 months), medium-length DAPT (6 months), and short DAPT (<6 months). Overall, no differences in mortality were observed between the durations. Extended DAPT had lower AMI, but no net benefit, except for patients with ACS and a low risk of bleeding. On the other hand, regarding ischemic events, reducing DAPT to 1 or 3 months was not inferior to 12 or 6 months. P2Y12 inhibitor monotherapy after a short period of DAPT provided the best net benefit for bleeding.^46^

This evidence provides a degree of security that, for time-sensitive noncardiac surgeries with a high risk of bleeding, DAPT can be shortened to 3 months or even 1 month. Although until recently it was recommended to wait 6 months between an elective PCI with a drug-eluting stent and a noncardiac surgery, 3 months can now be considered for new-generation stents. In more pressing situations, evidence already exists for discontinuing DAPT within 30 days, as is done for patients with non-drug-eluting stents. Before this period, only urgent or emergency noncardiac surgeries are justified ( Figure 1 ).

**Figure 1:** – Flowchart for defining the interval between noncardiac surgery and elective percutaneous coronary intervention. EL: evidence level

*Completely elective noncardiac surgical interventions should be performed after the optimal period of dual antiplatelet therapy.

For patients who underwent an acute PCI, the ideal is 1 year of DAPT before elective surgeries. However, when urgent procedures are required, DAPT can be reduced to 6 months and, exceptionally, to 1 month ( Figure 2 ).

**Figure 2:** – Flowchart for defining the interval between noncardiac surgery and percutaneous coronary intervention in the context of acute coronary syndrome.

*Completely elective noncardiac surgeries should be performed after the optimal period of dual antiplatelet therapy.

Due to the complexity of managing antithrombotic therapy in the perioperative period of noncardiac surgeries, the decision to shorten DAPT should consider the individual and surgical risk of thrombotic and hemorrhagic complications and should ideally be shared between the clinical cardiologist, the interventional cardiologist, and the surgical team.

### 2.1. Recommendations on the Interval between Elective Noncardiac Surgery and Percutaneous Coronary Intervention

## 3. Immediate Perioperative Care

### 3.1. Continue One Antiplatelet Agent

In all of these situations, only one antiplatelet agent should be removed, since removing both is associated with a shorter interval between DAPT modification and stent thrombosis^47^ – except for procedures with very high risk of bleeding, notably **neurosurgeries** , for which both antiplatelet agents should be interrupted and restarted as soon as possible. The recommendation is to continue with acetylsalicylic acid and discontinue the P2Y12 inhibitor a few days before the procedure, depending on the drug. In the case of DAPT with clopidogrel, it must be suspended 5 days before the procedure, with reintroduction after good hemostasis has been certified by the surgical team. Ideally, DAPT should not be suspended for more than 10 days perioperatively.^47^ When using ticagrelor, the recommendation is to suspend the drug 5 days before the procedure, despite the fact that platelet activity recovers faster than with clopidogrel.^7^ In the PLATO trial, this was corroborated by a sub-analysis of patients who underwent coronary-artery bypass grafting: among individuals randomized to acetylsalicylic acid + clopidogrel or ticagrelor, there was a lower bleeding rate in the group using new-generation P2Y12 inhibitors.^48^ Prasugrel must be discontinued 7 days before noncardiac surgeries.^49^

The PLAT-CABG trial assessed reducing the preoperative withdrawal time of P2Y12 inhibitors, using a platelet reactivity test before myocardial revascularization surgeries in patients who received acetylsalicylic acid + clopidogrel in the context of ACS. In this single-center Brazilian non-inferiority study, the normal 5-day presurgical discontinuation of clopidogrel was compared with using an adenosine diphosphate platelet reactivity test to guide the timing of surgery. The time required to perform the procedure was lower in the intervention group (112 hours vs 136 hours, p <0.001), and there was no impact on postoperative major bleeding events.^50^ Although there are still no specific recommendations on the systematic use of platelet aggregation tests, for more pressing situations when there is no surgical emergency but a clinical interest (based on individual assessment) in shortening the wait by 1 or 2 days, it may be worthwhile to consider such tests, provided common agreement with the surgical team.

The concept of “bridging therapy”, based on care for patients who use oral anticoagulation with warfarin, is reminiscent of the perioperative period of patients on DAPT. However, some differences are worth mentioning. Low-molecular-weight heparin does not effectively replace antiplatelet therapy and may even increase the incidence of hemorrhagic and thrombotic events.^51^ On the other hand, parenteral antiplatelet agents, which have a shorter half-life (glycoprotein IIb, IIIa inhibitors), were tested in a case series involving very high thrombotic risk,^52 , 53^ which is a class IIb recommendation according to current guidelines. We suggest only considering this for very special cases, especially when DAPT is interrupted less than 1 month after complicated PCI in an acute context ( Table 4 ).

**Table 4:** – Recommendations on the management of antiplatelet agents in the perioperative period

Summary of Recommendations	Recommendation class	Evidence level
Maintain acetylsalicylic acid at a dose of 100 mg per day throughout the perioperative period, except for neurosurgeries or procedures with prohibitive risk of bleeding.	I	A
Discontinue clopidogrel and ticagrelor 5 days before noncardiac surgery.	I	B
Discontinue prasugrel 7 days before noncardiac surgery.	I	B
If the ideal minimum period of DAPT cannot be completed, perform noncardiac surgeries in centers with multidisciplinary and hemodynamic support.	I	C
Perform surgeries with a low risk of bleeding during DAPT, if the time since angioplasty is <3 months.	IIa	C
Use a platelet aggregability test to shorten P2Y12 inhibitor discontinuation time before noncardiac surgery.	IIb	B
For cases with very high thrombotic risk (<1 month after PCI and DAPT interruption), use tirofiban as a bridging therapy.	IIb	B
Use low-molecular-weight heparin for bridging therapy.	III	B

Thus, research and technological advances are moving towards shortening DAPT, which, above all, benefits patients at high risk of bleeding. However, whenever DAPT is interrupted earlier than planned, the surgery must be performed in a center with multidisciplinary support, including cardiovascular monitoring and hemodynamic backup, in case there are complications.

## 4. Prophylactic Myocardial Revascularization

Debate about the ideal interval between PCI and noncardiac surgery may arise when a patient with a recent PCI requires surgery or when considering myocardial revascularization during preoperative assessment of cardiac risk. Randomized studies have found that prophylactic myocardial revascularization has no significant impact on the occurrence of ischemic events in patients with planned vascular surgery.^54 - 56^ Thus, myocardial revascularization is recommended only in individuals with an unequivocal indication for the procedure, regardless of perioperative context. It should not be routinely performed just to reduce perioperative cardiovascular complications. In these cases, decision-making must always consider the patient’s clinical context, the prognosis of the underlying disease, the minimum DAPT period for PCIs, and the risk of bleeding associated with the intervention. It should be reiterated that a predicted noncardiac surgery should not lead to a preference for non-pharmacological stents: this concept no longer seems viable in light of the new evidence. Another possibility for situations in which PCI is indicated due to a very high ischemic risk and a time-sensitive noncardiac surgery is required would be performing PCI with a balloon alone and no stent. Little evidence has been published about such a strategy apart from aspirin monotherapy and an interval of at least 2 weeks until the operation.^57^ There is no guarantee at the planning stage that this will be feasible, since stenting may be necessary to ensure the primary outcome of the intervention. Thus, this strategy should not be routinely used.

Specific indications for myocardial revascularization should follow chronic and acute coronary disease guidelines.^58 , 59^
